# Global expression of *AMACR* transcripts predicts risk for prostate cancer – a systematic comparison of AMACR protein and mRNA expression in cancerous and noncancerous prostate

**DOI:** 10.1186/s12894-016-0128-8

**Published:** 2016-02-29

**Authors:** Saeid Alinezhad, Riina-Minna Väänänen, Natalia Tong Ochoa, Emily A. Vertosick, Anders Bjartell, Peter J Boström, Pekka Taimen, Kim Pettersson

**Affiliations:** Division of Biotechnology, University of Turku, Tykistökatu 6A 6th floor, 20520 Turku, Finland; Department of Epidemiology and Biostatistics, Memorial Sloan-Kettering Cancer Center, New York, NY USA; Department of Clinical Sciences, Division of Urological Cancers, Lund University, Skåne University Hospital, Malmö, Sweden; Department of Urology, Turku University Hospital, Turku, Finland; Department of Pathology, University of Turku and Turku University Hospital, Turku, Finland

**Keywords:** Prostate cancer, mRNA expression, Biomarker, AMACR, Radical prostatectomy and cystoprostatectomy

## Abstract

**Background:**

The high false negative rates for initial prostate biopsies refer a large number of the men for repeat biopsies each year. Therefore, biomarkers associated with high risk of the presence of malignancy in histologically benign biopsies could provide a tool to discriminate the patients who need repeat biopsy or intensive follow-up from those who do not. Here we examined the diagnostic applicability of alpha-methylacyl CoA racemase (*AMACR)* and androgen receptor (*AR)* mRNA expression and AMACR protein levels in benign and cancerous prostatic tissue.

**Methods:**

*AMACR* and *AR* mRNA levels were measured with quantitative, reverse-transcription PCR (qRT-PCR) assays in 79 radical prostatectomy (RP) cases (including 69 benign (RP-Be) and 69 cancerous (RP-PCa) samples) and 19 benign prostate samples obtained from cystoprostatectomies. To further determine the detailed areas of altered *AMACR* expression*, AMACR* mRNA level measurement and protein staining were performed for three cross-sectioned RP cases.

**Results:**

The median *AMACR* and *AR* expression levels were 194.6 (*p* < 0.0001) and 6.6 (*p* = 0.0004) times higher in RP-PCa samples than in the benign cystoprostatectomy (CP) samples, respectively. There was no statistically significant difference between RP-PCa and RP-Be samples, except for *AMACR/KLK3* (Kallikrein-Related Peptidase 3) ratio, which was significantly higher in RP-PCa samples than in RP-Be samples (*p* = 0.016). In the systematic study of cross-sections, *AMACR* mRNA was detected in all of the studied areas including histologically benign tissue, but at significantly higher levels in carcinoma areas (*p* < 0.001). AMACR protein expression was detected in 80 % (28/35) of the areas that contained carcinoma and in 37 % (44/119) of the benign and PIN areas from the same patients.

**Conclusions:**

*AMACR* transcripts were detected in all RP-PCa and RP-Be samples but not in non-cancerous CP samples, which suggest a global increase of *AMACR* expression in cancerous prostates. Therefore patients with false negative biopsies might benefit from an *AMACR* mRNA measurement when assessing their cancer risk.

## Background

Prostate cancer (PCa) is the most common cancer in men and the second leading cause of cancer-related death. The current routine diagnostic approach employs a combination of digital rectal examination (DRE) and measurement of serum level of prostate specific antigen (PSA) followed by histological examination of prostate biopsies. However, negative biopsy results have been reported for 70–80 % of the men in the United States who undergo prostate biopsy each year [1]. Considering the fact that in those men, cancer foci might have been missed due to sampling error, repeated biopsies for cases with elevated PSA level are an assuring option. Re-biopsies or increasing the number of biopsy cores have been shown to improve the diagnostic accuracy and reduce false negative results [2], but the procedures are costly and generate discomfort for patients. Therefore, biomarkers associated with high risk of presence of malignancy on histologically benign biopsies could provide an additional tool in identifying those patients who would truly benefit from a repeat biopsy or intensive follow-up.

Extensive research based on high-throughput gene expression profiling has aimed to identify new biomarkers in PCa tissues to better address the limitations of present diagnostic and prognostic approaches. Here we performed validation of diagnostic capabilities of quantitative measurement of alpha-methylacyl CoA racemase (*AMACR*) and androgen receptor (*AR*) transcripts by RT-PCR for PCa diagnosis.

AMACR enzyme is involved in peroxisomal beta-oxidation of branched-chain fatty acids and their derivatives [3]. In PCa *AMACR* is highly expressed at both mRNA and protein levels [4]. However, *AMACR* is also frequently expressed in prostatic intraepithelial neoplasia (PIN) [5] and variable expression may be found in benign prostatic glandular epithelium across all age groups [6]. Therefore, to resolve diagnostic challenges in clinical pathology AMACR staining is typically combined with a basal epithelial cell marker such as p63 or 34βE12 that are not present in PCa.

The growth and maintenance of prostate tissue is dependent on androgens produced in testes and adrenal glands [7] and the intracellular androgen receptor (*AR*) signaling plays a key role in the formation and development of PCa. Androgen-ablation therapy is one of the most common and successful treatments of PCa but with time the tumor frequently reaches an androgen-independent state. One mechanism for PCa to become castration resistant is due to the amplification and overexpression of *AR* gene enabling the cancer cells to grow with very low levels of androgen [8, 9]. Thus, overexpression of *AR* promotes the formation and progression of PCa as well as castration resistant PCa.

Overexpression of *AMACR* - at both mRNA and protein levels - has been reported in cancerous prostatic tissue when compared to benign prostatic hyperplasia (BPH) and normal prostate tissue [4, 10–14]. The aim of our study was to investigate the potential use of *AMACR* and *AR* mRNA expression levels to improve detection of PCa. We examined the mRNA levels of *AMACR* and *AR* in 157 prostate tissues by using internally standardized, truly quantitative reverse-transcription PCR (qRT-PCR) assays. The samples were collected from 79 cases of radical prostatectomies (RP) rapidly after the procedure both from the suspected tumor sites and from areas away from the tumor site. Cystoprostatectomy derived samples (CP) from 19 non-PCa patients or patients harboring incidental PCa were used as controls. All cystoprostatectomies were performed because of bladder cancer with no clinical suspicion of PCa. Based on the initial, *AMACR*-related findings of these experiments, we also set out to perform a preliminary, systematic evaluation of whole prostate cross-sections from three PCa patients to determine if samples from areas outside the pathologically determined tumor lesions could be equally informative of the presence of cancer. *AMACR* expression in these cross-sections was examined on both mRNA and protein levels by qRT-PCR and immunohistochemistry, respectively, and compared to the tissue morphology.

## Methods

### Radical prostatectomy and cystoprostatectomy samples

The study protocol was approved by the Ethics Committee of the Hospital District of Southwest Finland and it was in accordance with the Helsinki Declaration of 1975, as revised in 1996, with written informed consent obtained from each participant.

In total, 157 prostate tissue samples were examined. The cohort consisted 79 RP patients (Table 1) from whom 138 tissue samples containing 69 histologically benign samples (RP-Be) and 69 cancerous samples (RP-PCa) were obtained after radical prostatectomies in Turku University Hospital in Turku, Finland for clinically localized PCa. (Table 2 represents clinicopathological data for 138 samples obtained from 79 radical prostatectomy specimens). Two tissue samples were collected from each prostate: one from an area suspected to contain cancer and one from a macroscopically normal area as a control [15]. Pathological examination revealed that for some patients both samples had been taken from either benign or cancerous areas. For 59 prostates we examined both samples (for 30/59 prostates one of the samples had been taken from a benign area and the other from a cancerous area; for 15/59 both samples were from a cancerous area; and for 14/59 both samples were from a benign area), and for 9 and 11 prostates, we only had access to one sample per prostate, similarly taken either from the cancerous or control area, respectively. The samples were collected immediately after the removal of the organ and stored in quanidine isothiocyanate buffer [15]. The final content of the samples (benign or cancerous) was determined from hematoxylin-eosin (HE) stained frozen sections obtained next to the fresh frozen tissue samples.

**Table 1:** Patients characteristics

Number of patients	79	
Age at surgery (years) Average (min,max)	61.5 (48, 71)	
Preoperative serum PSA Average (min,max)	8.4 (1.4, 30)	
Pathological T-category	Number of samples (percentage)	
	pT2	33 (42 %)
	pT3 and pT4	41 (52 %)
	Unknown	5 (6 %)
Pathological Gleason score		
	≤6	45 (57 %)
	7	18 (23 %)
	≥8	12 (15 %)
	Unknown	4 (5 %)

**Table 2:** Clinicopathological data for 138 samples obtained from 79 radical prostatectomy specimens

	Number of samples (percentage)
Pathological T-category	
pT2	57 (41 %)
pT3 and pT4	72 (52 %)
Unknown	9 (7 %)
Pathological Gleason score	
≤ 6	81 (58 %)
7	27 (20 %)
≥ 8	22 (16 %)
Unknown	8 (6 %)

Additionally, 19 histologically benign prostatic tissue samples were obtained from patients who underwent cystoprostatectomy due to bladder cancer without clinical evidence of PCa in Skåne University Hospital in Malmö, Sweden. At the time of histological evaluation, seven cases showed no evidence of PCa (CP-Be) while 12 had incidental PCa (CP-PCa)).

### Prostate cross-section samples

Single whole prostate cross-sections were obtained from three patients with localized PCa who underwent RP in Turku University Hospital in Turku, Finland. The cohort and sample collection has been previously reported [16]. Briefly, a 2 mm horizontal mid-plane tissue slice covering the entire gland was obtained from each prostate for the experiment. To be able to have a unique coordinate code for each piece of tissue, the prostate slice was fixed on a Styrofoam plate with a 5 × 5 mm grid guide on it. To avoid cross-contamination, sterile blades were used to cut the prostate slices further into 5 × 5 × 2 mm pieces. Based on the size of the prostate gland, 48, 62 and 44 pieces of tissue were obtained fromprostates A, B and C, respectively. The samples were stored in RNAlater (Qiagen) at −20 °C until RNA extraction.

For histological examination, from each specimen, the tissue adjacent to the slice used for mRNA measurements were fixed in formalin and embedded in macro paraffin blocks (FFPE). The FFPE blocks covering the superior and inferior side of the cross-section were cut to 5 μm sections and HE-stained.

### RNA isolation and reverse transcription

Total RNA was extracted from the tissues and reverse transcribed to cDNA as previously described [15]. During the extraction, a known amount of RNA of artificially mutated *KLK3* gene called mmPSA [17] was added to samples as internal standard after the cell lysis.

### Real-time PCR

A previously described concept utilizing target-specific oligonucleotide probes and time-resolved fluorometry were used to detect the accumulation of *AMACR* and *AR* transcripts [18, 19]. Oligonucleotide primers and probes (Table 3) were purchased from Thermo (Germany). All samples were run in triplicate in a reaction volume of 25 μl containing 2.5 μl of template cDNA using a previously described temperature profile [19] and external DNA standards [15].

**Table 3:** The oligonucleotides used in this study

Oligonucleotide	Sequence (5′ to 3′)	Position^a^	Lanthanide chelate label or quencher molecule
*AMACR* forward primer	TTGTCAGGTGTTCTCTCAAA	481–500	
*AMACR* reverse primer	CTTCCACCATATTTGCATC	637–655	
*AMACR* reporter probe	C^b^TGAATCTCCTGGCTGACTTTGCTGG	535–560	9d-α gal-Eu^III^
*AMACR* quencher probe	TCAGCCAGGAGATTCAG^c^	535–551	Dabcyl
*AR* forward primer	GCTGAAGGGAAACAGAAGTAC	328–348	
*AR* reverse primer	CTCTCCTTCCTCCTGTAGTTTC	480–501	
*AR* reporter probe	T^b^TGTCGTCTTCGGAAATGTTATGAAGCAGG	408–437	9d-α gal-Eu^III^
*AR* quencher probe	AACATTTCCGAAGACGACAA^c^	408–427	Dabcyl

^a^gene bank accession numbers NM_014324 and NM_001011645 were used for *AMACR* and *AR* nucleotide sequences, respectively

^b^internally amino-modified C6-base for labeling with Eu chelate

^c^quencher molecule

### Immunohistochemistry

For cross-section studies, histological macrosections of 5 μm in thickness were cut from the FFPE blocks of each prostate (A,B and C), next to the HE-stained sections. Paraffin was removed with xylene and the sections were rehydrated with series of alcohol. Antigen retrieval was carried out by microwaving the slides in Target Retrieval solution (Dako) at pH 9 for 7 min. The tissue sections were incubated for one hour with rabbit monoclonal AMACR (P504S) antibody (1:200, clone 13H4, Zeta Corporation). The primary antibody was detected with EnVision + Dual Link System-HRP (Dako) and visualized with DAB+ chromogen solution (Dako). The slides were observed by an experienced uropathologist using a Leica DM3000 light microscope equipped with Leica DFC 420 digital camera and Leica Application Suite version 2.5.0 R1 (Leica Microsystems, Wetzlar).

### Data analysis

The obtained mRNA levels were normalized to total RNA amount and the previously determined internal RNA standard levels. Samples were considered as positive only when all three replicates were positive and above the lowest detection limit of the assay. To calculate the expression levels of *AMACR* or *AR* in relation to *KLK3* (the gene encoding PSA), the mRNA copy numbers were divided by previously determined *KLK3* mRNA levels [20].

Linear regression models were created to test whether the type of sample (RP-BE, RP-PCa, CP-Be, CP-PCa) was associated with the log-transformed expression levels of *AR*, *AMACR*, *KLK3*, *AR/KLK*3 or *AMACR/KLK3*. Mann–Whitney *U* test was used to evaluate whether there were statistically significant differences in the gene expression levels between any two of the groups. A significance level of *p* = 0.05 was used.

For the systematic study of cross-sections, the histology of tissue samples used for mRNA expression measurements was defined by dividing the digital images of HE-stained tissue slides into equal amount of regions with the cross-sections used in mRNA experiments. Each sample piece was given coordinates on two axes (one axis got values from A to K and the other axis from 1 to 10). Based on histopathological examination, the samples were categorized into three groups: carcinoma (*n* = 35), if one or both adjacent HE-stained sections revealed adenocarcinoma at that location; histologically benign tissue (*n* = 112), if that is what both HE-stained sections contained; and PIN (*n* = 7), if one or both HE-stained sections contained PIN lesions but no carcinoma.

## Results

### *AMACR, AR* and *KLK3* mRNA expression in RP and CP samples

For the PCa patients, the median, minimum and maximum age and preoperative PSA level in serum were 63 (48 and 71) years and 6.95 (1.4 and 30) ng/ml, respectively. Detailed clinicopathological data of the patients are shown in Table 1.

**Fig. 1:**
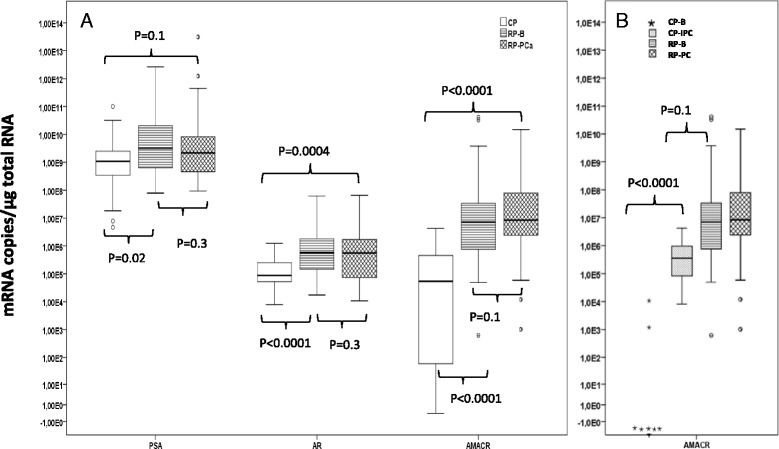
**a** *KLK3*, *AR* and *AMACR* mRNA levels in cystoprostatectomy (CP) samples (with and without incidental PCa), histologically benign radical prostatectomy (RP-Be) samples and cancerous radical prostatectomy (RP-PCa) samples. **b** *AMACR* mRNA levels in CP samples without incidental PCa (CP-Be), CP samples with incidental PCa (CP-PCa), RP-Be and RP-PCa samples. The top whisker represents highest case within 1.5 times IQR. The top line of the box represents the 3rd quartile, the line in the middle of the box represents the median, the bottom line of the box represents 1st quartile and bottom whisker represents the lowest case within 1.5 times IQR. Open circles represent the outlier values

The limit of detection for the *AMACR* assay was 10 copies/μl of template DNA and 1 copy/μl of template DNA for the *AR* and *KLK3* assays. *AMACR* mRNA was detected in all RP-Be, RP-PCa and CP-PCa samples but only in 2 out of 7 CP-Be tissue samples (Fig. 1a). All tissue samples were found to contain detectable levels of *AR* and *KLK3* transcripts. Table 4 represents the mRNA expression level of target genes in different group of samples.

**Table 4:** The mRNA expression level (copies/μg of total RNA) of target genes in different group of samples

	Expression level (copies/μg of total RNA)			
Target genes	CP-Be (*n* = 7)	CP-PCa (*n* = 12)	RP-Be (*n* = 69)	RP-PCa (*n* = 69)
*KLK3*	1 × 10^9^ (4.6 × 10^6^, 3.2 × 10^10^)	1.2 × 10^9^(7.8 × 10^6^, 9.9 × 10^10^)	3.2 × 10^9^(7.8 × 10^7^,2.6 × 10^12^)	2.1 × 10^9^(9.9 × 10^7^,3.1 × 10^13^)
*AMACR*	0 (0, 2.7 × 10^4^)	3.5 × 10^5^(8.1 × 10^3^,4.2 × 10^6^)	7 × 10^6^(6.2 × 10^2^,4.1 × 10^10^)	8.3 × 10^6^(1 × 10^3^,1.5 × 10^10^)
*AR*	5.1 × 10^4^ (7.8 × 10^3^, 1,2 × 10^6^)	1 × 10^5^(4.4 × 10^4^,1.1 × 10^6^)	5.6 × 10^5^(1.7 × 10^4^,6.2 × 10^7^)	5.5 × 10^5^(1 × 10^4^,6.5 × 10^7^)

Data are presented as median (min, max). CP-Be represents cystoprostatectomy samples with no evidence of PCa, CP-PCa represents cystoprostatectomy samples with incidental PCa, RP-Be represent histologically benign samples from radical prostatectomy specimens and RP-PCa represents cancerous samples from radical prostatectomy specimens

Compared to the 19 CP samples, the median *AMACR* expression level was 126 times higher in the RP-Be samples (*p* < 0.0001) and 195 times higher in the RP-PCa samples (*p* < 0.0001) (Fig. 1). When the median *AMACR* expression level in the RP-PCa samples was compared to the mean value of the two *AMACR* positive CP-Be samples there was a 682-fold difference (*p* < 0.0001). There was no statistically significant difference in *AMACR* mRNA levels between the RP-PCa and RP-Be groups. Comparison of AMACR expression level by paired *t*-test between cancerous and benign samples for 30 patients who had two matched samples resulted in insignificant difference (*P* > 0.05). However, the *AMACR* to *KLK3* mRNA ratio was 5.3 times higher in the RP-PCa than in the RP-Be group (*p* = 0.012).

In comparison to all of the 19 CP samples, the median level of *AR* expression was 6.4 times higher in the RP-Be samples (*p* < 0.0001) and 6.6 times higher in the RP-PCa samples (*p* = 0.0004) (Fig. 1). The greatest difference in median levels of *AR* expression was found between the RP-PCa samples and the CP-Be samples (11.5-fold, *p* = 0.004). No statistically significant differences were seen in *AR* or *KLK3* expression, nor in *AR*/*KLK3* mRNA ratio between the RP-PCa and RP-Be samples.

The mRNA levels of the target genes were not associated with Gleason grade in the RP samples, but the expression levels of target genes were statistically significantly higher in samples from men with either stage pT3 or pT4 tumors (*n* = 72) than in samples from men with PCa classified as pT2 (*n* = 57) (*AMACR*, *p* = 0.006; *AR*, *p* = 0.005 and *KLK3*, *p* = 0.004).

Based on Pearson’s correlation coefficient, there was a strong correlation (*r* = 0.86) between *AR* and *AMACR* expression levels when samples from all groups were combined. Furthermore there was no strong correlation between *AMACR* or *AR* and serum PSA (*R* = 0.46 and *R* = 0.18, respectively).

**Fig. 2:**
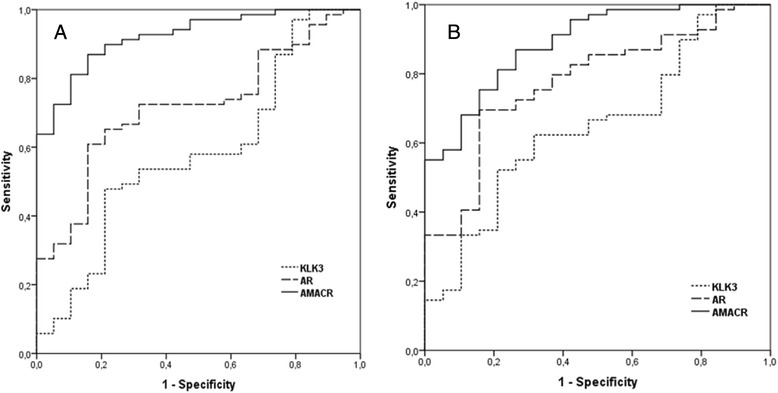
ROC curve analyses for *KLK3*, *AR* and *AMACR* mRNA expression. **a** ROC curve analysis for RP-PCa and CP tissues. AUC values are 0.58, 0.717 and 0.923 for *KLK3*, *AR* and *AMACR* mRNA levels, respectively. **b** ROC curve analysis for RP-Be and CP tissues. AUC values are 0.64, 0.77 and 0.89 for *KLK3*, *AR* and *AMACR* mRNA levels, respectively

The receiver operating characteristic (ROC) curve analyses for *AMACR*, *AR* and *KLK3* mRNA expression for evaluating the diagnostic potency and discrimination power of the target genes between all RP samples and CP samples produced area under the curve (AUC) values of 0.94, 0.74 and 0.61, respectively. The ROC curve analyses for *AMACR*, *AR* and *KLK3*mRNA expression for evaluating the correlation of the expression level with histology between RP-PCa and CP samples produced AUC values of 0.923, 0.717 and 0.58, respectively (Fig. 2a). Corresponding AUC values for RP-Be specimens were 0.89, 0.77 and 0.64, respectively (Fig. 2b).

### Systematic analysis of *AMACR* mRNA and protein expression in prostate tissue cross-sections

**Fig. 3:**
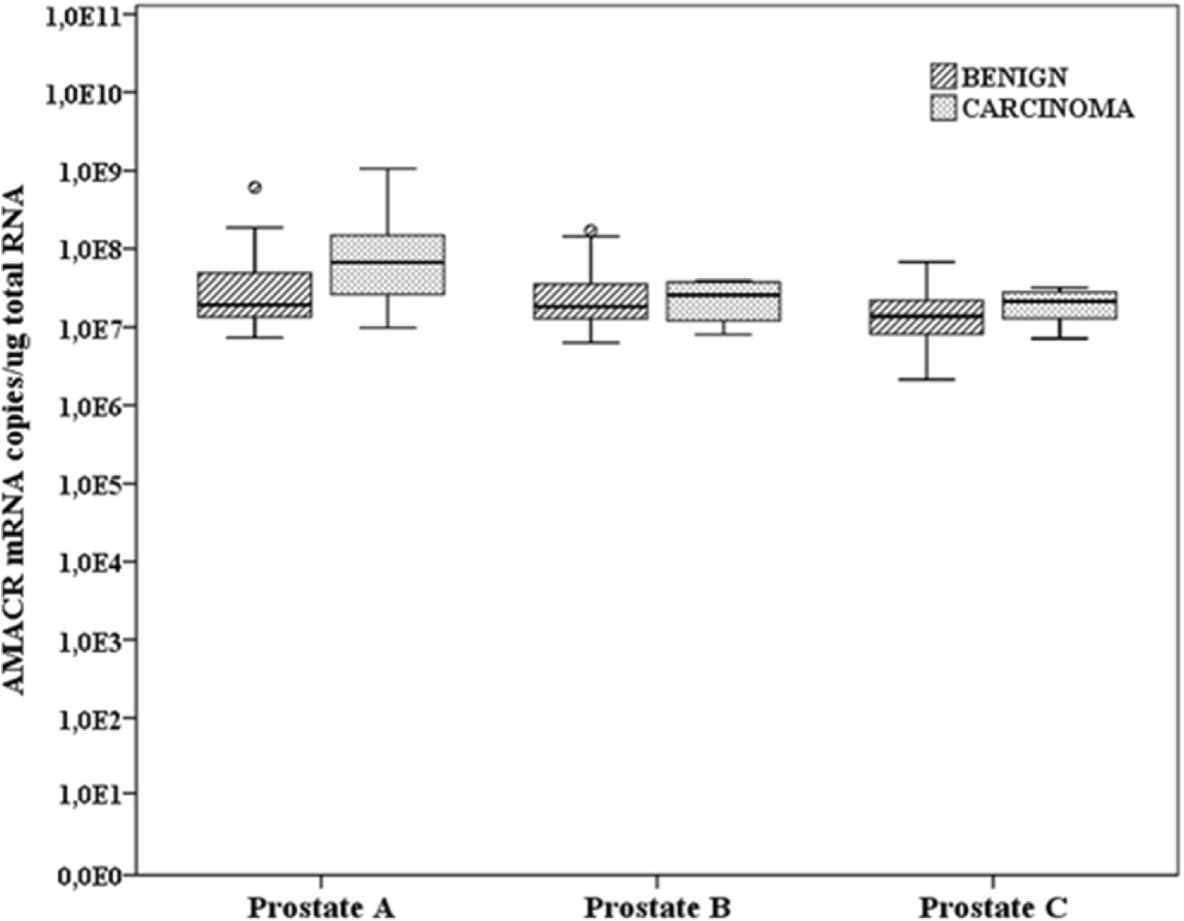
*AMACR* mRNA expression levels in histologically benign and cancerous (carcinoma) samples from three prostate cross-sections obtained from three patients with localized PCa who underwent RP. The 10/25/50/75/90th percentiles are marked in the figures. Open circles represent the outlier values

*AMACR* mRNA was universally expressed in all tissue pieces covering the whole cross-section of prostates A, B and C included in the analysis. As a comparison, all measured values from the cross-section samples were above the highest expression level seen in CP-Be samples (Fig. 3). There was a significant, 1.9-fold difference (*p* < 0.001) in median *AMACR* mRNA levels between carcinoma samples (*n* = 35) and histologically benign (*n* = 112) samples. There was no significant difference between PIN samples (*n* = 7) and either carcinoma or histologically benign samples.

**Fig. 4:**
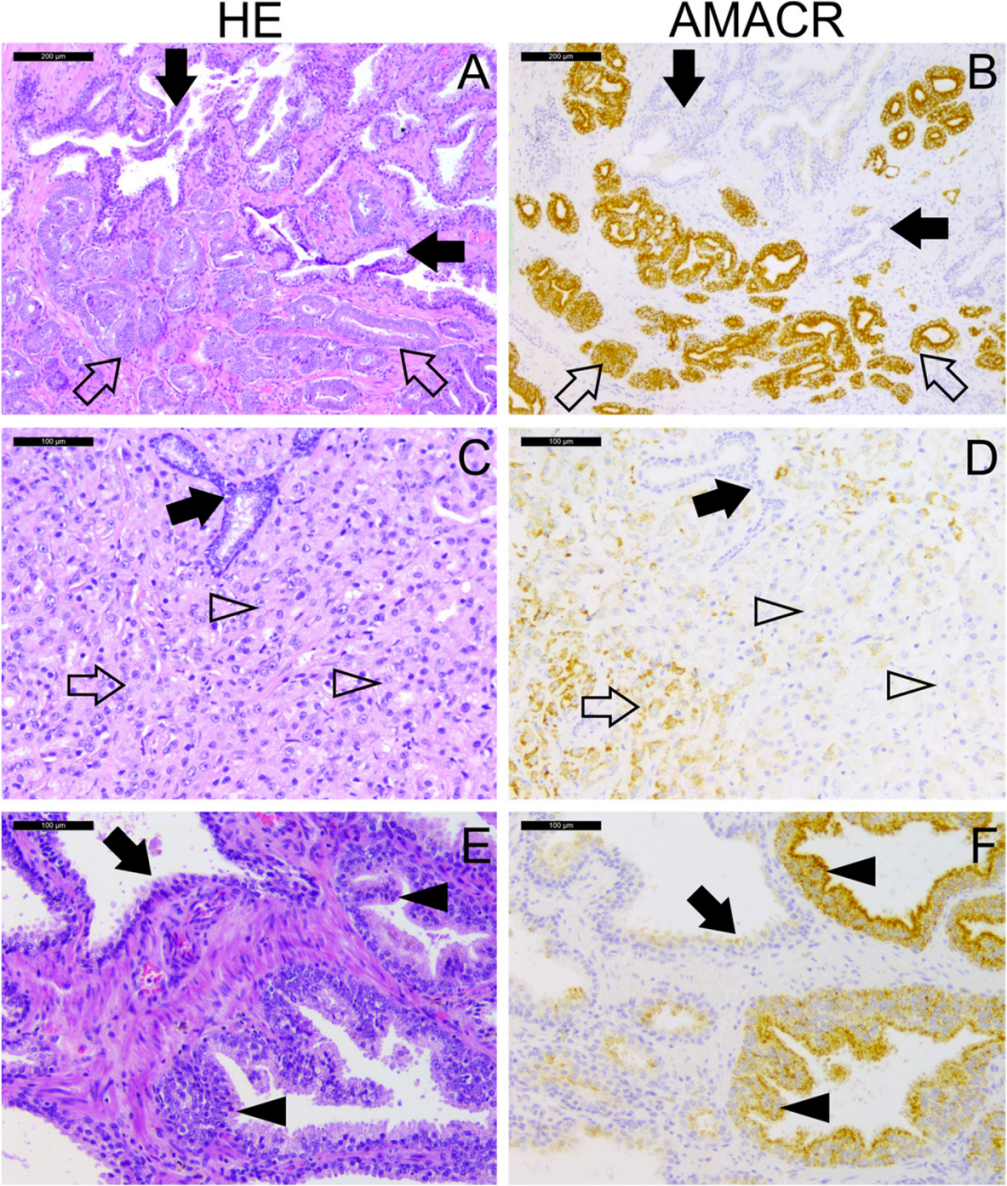
Immunohistochemical analysis of AMACR protein expression in different prostatic lesions. Hematoxylin-eosin (HE) and AMACR staining from the same region of interest are shown. **a**, **b** Typical acinar Gleason 3 + 4 adenocarcinoma stains strongly for AMACR (*open arrow*) while the normal glands are AMACR negative (*filled arrow*). **c**, **d** Gleason 4 + 5 adenocarcinoma shows focal positive staining for AMACR (*open arrow*) while some of the poorly differentiated carcinoma glands (*open arrowhead*) and normal glands (*filled arrow*) are AMACR negative. **e**, **f**: High grade PIN lesion shows positive AMACR staining (*filled arrowhead*) while morphologically normal glands show weak or no AMACR staining (*filled arrow*). Scale bar 200 μm (**a**-**b**), 100 μm (**c**-**f**)

In the immunohistochemical analysis (Fig. 4), AMACR protein was detected in all of the three prostate cross-sections. More specifically, AMACR staining was positive in 100 % of areas that contained carcinoma in both superior and inferior sides of cross-sections from prostate B and C. However on the superior side of prostate A, AMACR staining was positive in 96 % of the carcinoma areas while some Gleason 4 + 5 carcinoma foci were found virtually AMACR negative (Fig. 4c-d). Strong positive AMACR staining was constantly observed in prostatic intraepithelial neoplasia (PIN) lesions (Fig. 4e-f). While the great majority of normal prostatic glands were AMACR negative, we occasionally observed some weak AMACR staining in morphologically benign glands especially adjacent to carcinoma lesions. However, the staining in such glands was virtually significantly weaker when compared to that seen in carcinoma and PIN lesion. These results suggest that AMACR mRNA is globally expressed in cancerous prostates while AMACR protein is most abundant in carcinoma and PIN lesions.

## Discussion

Each year a large number of the men undergo repeat biopsy due to elevated PSA level. Repeat biopsy results have demonstrated high rates of false negative results of initial prostate biopsy (10 % to 30 %) [21, 22]. The biopsy procedure is expensive and carries several risks and discomfort for the patients. Therefore, finding reliable molecular markers that would enable early and accurate diagnosis of PCa is of importance.

Numerous studies based on the measurement of mRNA and/or protein expression have reported the upregulation of a variety of markers in PCa such as *AMACR* and *AR*. Based on several previous immunohistochemistry studies, AMACR is used as a routine tissue biomarker to support the diagnosis of prostate cancer [4, 12, 23, 24] and especially useful for detection of small carcinoma foci in needle biopsies when combined with basal cell marker such as keratin 5/6 or p63 [12]. Furthermore, AMACR immunocytochemistry and microscopic evaluation of cells captured from post-DRE urine has been applied for detection of PCa [25]. However, there are relatively few studies on *AMACR* or *AR* mRNA expression levels in prostate cancer tissue [26, 27]. Against this background, we measured the mRNA expression levels of *AMACR* and *AR* in 157 malignant and histologically benign prostate tissues with qRT-PCR assays.

We observed an overexpression of both *AMACR* and *AR* mRNA in not only cancerous, but also in histologically benign tissue from prostates with clinically significant carcinomas when they were compared to prostates without tumor or harboring only incidental lesions. Both mRNAs were also positively associated with advanced pathologic stage. The detected overexpression in cancerous tissues was 682-fold for *AMACR* mRNA and 11.5-fold for *AR* mRNA compared to prostates without any evidence of cancer. However, no differences in gene expression were seen when cancerous and histologically benign tissues from PCa patients were compared, except for *AMACR/KLK3* mRNA ratio. This supports the finding in a previous study by Laxman et al. that *AMACR* as a single marker does not have sufficient sensitivity and specificity and that it could provide better detection of PCa only in a multiplex setting [28].

Finding abnormal *AMACR* levels in benign tissues motivated us to examine the alteration in *AMACR* mRNA levels and protein expression in a systematic way in cross-sections of whole cancerous prostates. On the protein level, expression of AMACR is sometimes detected in benign biopsies from patients with PCa, as well as in normal glands that were in closer proximity to a carcinoma foci [5]. Similar results we obtained in the present study (Fig. 4). The phenomenon of increased biomarker expression in histologically benign tissue in itself can be due to cancer field effect [29, 30]. Field cancerization or field effect which was suggested by Slaughter et al. in 1953 [31] has an extended definition nowadays and it defines as any molecular abnormalities in tissues that appear histologically benign [32]. We found that in contrast to the carcinoma-free CP specimens where *AMACR* expression was rare, *AMACR* mRNA was detectable in all benign, carcinoma and PIN samples obtained from the three studied prostate cross-sections with highest levels in carcinoma samples. As expected, AMACR staining was detected in the carcinoma areas, but not in all of them, and additionally, it was seen in some of the benign and PIN areas. As expected, AMACR staining was strongly positive in the great majority (but not all) carcinoma and PIN lesions. In additionally, we occasionally observed variable AMACR positivity in morphologically benign glands either adjacent to or separated from the cancerous foci showing that AMACR expression is not specific for PCa. Accordingly, measurement of the AMACR protein in urine samples by ELISA has not been able to discriminate between BPH and PCa better than serum PSA suggesting that AMACR may also be expressed under reactive conditions such as hyperplasia

In addition to the naturally occurring variable expression in benign glands [5], the difference between mRNA and protein level may be at least partly due to sensitivity limitations and differences in antibodies and detection systems [10, 13, 23]. Another possibility is the fact that the experimental set-up used here is unable to specifically account for the tissue shrinkage during fixation, which may affect the accuracy in defining the exact sample areas that correspond to the sample pieces in mRNA measurements and thereby affect especially the data obtained from samples adjacent to carcinoma foci. In this study we used samples from men with Caucasian ethnicity, but previously lower sensitivity and specificity of AMACR for the diagnosis of PCa has been reported for example in Japanese patients [33]. The involvement of AMACR in peroxisomal beta-oxidation of branched-chain fatty acids and the differences in diet habits between western and Japanese men could be considered as one explanation but extra validation using patient cohorts from different ethnic origins are needed to fully understand the differences in AMACR expression in the future. Nevertheless, the combined mRNA and protein data from the cross-sections could suggest that qRT-PCR assays are more sensitive than immunohistochemistry to reveal potential carcinoma lesions, because in accordance with our results for carcinoma samples in immunohistochemical analysis, it has been reported that some small foci of prostate cancers can be negative for AMACR staining [34]. One limitation of this study was using fresh frozen material while FFPE material is used in routine practice. The positive aspect of using fresh frozen, rapidly processed and stored in RNA stabilization reagent is that it clearly approaches the in-vivo situation. Using FFPE samples for gene expression studies are challenging due to RNA degradation and modification during the embedding and fixation. Gene expression analysis of AMACR and AR in FFPE samples using novel technologies such as nCounter (NanoString® Technologies, USA) could be considered as further validation in future.

## Conclusion

Similar expression of AMACR transcripts in all of the RP-Be and RP-PCa samples suggests that AMACR expression is increased also in the histologically benign areas of cancerous prostates. This could potentially indicate a global overexpression of AMACR as was detected in the preliminary yet systematic cross-section study here. Hence, it could be deduced that patients with false negative biopsies (i.e., lesion missed in the biopsies) might benefit from an AMACR mRNA measurement with a qRT-PCR assay when assessing their cancer risk. Although this study was limited in the number of control tissue samples from PCa-free patients, but these data poses interesting options for further studies. In the future, this assay could also be applied to different sample matrices such as urine samples in order to move towards developing a noninvasive assay.

## Abbreviations

AMACR: alpha-methylacyl CoA racemase
AR: androgen receptor
CP: cystoprostatectomy
CP-Be: benign cystoprostatectomy samples
CP-PCa: cystoprostatectomy samples with incidental PCa
CRPC: castration resistant prostate cancer
BPH: benign prostatic hyperplasia
HE: hematoxylin-eosin
KLK3: kallikrein-related peptidase 3
PCa: prostate cancer
PIN: prostatic intraepithelial neoplasia
PSA: prostate specific antigen
qRT-PCR: quantitative reverse transcription polymerase chain reactions
RP: radical prostatectomies
RP-Be: histologically benign radical prostatectomy samples
RP-PCa: cancerous radical prostatectomy samples
